# Threshold Switching of Ag-Ga_2_Te_3_ Selector with High Endurance for Applications to Cross-Point Arrays

**DOI:** 10.1186/s11671-021-03585-0

**Published:** 2021-08-09

**Authors:** Jaeyeon Kim, Jimin Lee, Minkyu Kang, Hyunchul Sohn

**Affiliations:** Department of Materials Science and Engineering, Yonsei University, 03722 Seoul, Republic of Korea

**Keywords:** Thin films, Vapor deposition, Diffusion, Electronic properties, TEM

## Abstract

Threshold switching in chalcogenides has attracted considerable attention because of their potential application to high-density and three-dimensional stackable cross-point array structures. However, despite their excellent threshold switching characteristics, the selectivity and endurance characteristics of such selectors should be improved for practical application. In this study, the effect of Ag on the threshold switching behavior of a Ga_2_Te_3_ selector was investigated in terms of selectivity and endurance. The Ag-Ga_2_Te_3_ selector exhibited a high selectivity of 10^8^ with low off-state current of < 100 fA, steep turn-on slope of 0.19 mV/dec, and high endurance of 10^9^ cycles. The transient response was verified to depend on the pulse input voltage and measurement temperature. Considering its excellent threshold switching characteristics, the Ag-Ga_2_Te_3_ selector is a promising candidate for applications in cross-point array structures.

## Introduction

Resistive random-access memory has been investigated as a promising candidate for next-generation nonvolatile memory, owing to its simple operation, low power consumption, three-dimensional (3D) stackable potential, scalability, and simple structure [1–4]. However, the sneak current passing through adjacent cells must be reduced to avoid the potential operation failure that can occur in 3D cross-point array (CPA) structures with high cell density [5, 6]. Two-terminal selector devices with low off-state currents and high on/off ratios are favored to address such sneak current issues [7, 8].

Various types of selector devices with threshold switching (TS) characteristics have been proposed previously, including Ovonic threshold switch (OTS) [9], metal−insulator transition (MIT) [10], field-assisted super-linear threshold switch (FAST) [11], electrochemical metallization (ECM) [12], and mixed-ionic-electronic conduction (MIEC) [13]. However, the selectivity and leakage current of OTS and MIT selectors should be improved for practical applications [9, 10]; the nature of materials used for FAST selectors is not known [11]. Meanwhile, ECM and MIEC devices with Ag or Cu have attracted considerable attention because of their desirable TS characteristics, including their low leakage current, high on/off ratio, steep turn-on slope, and large hysteresis between the threshold voltage (*V*_TH_) and hold voltage (*V*_Hold_) [14–16]. In a one-selector-one resistor (1S1R) structure, the voltage window for the read operation is determined by the set voltage (*V*_Set_) of the memory and *V*_TH_ of the selector. Because *V*_Set_ varies according to the materials used for the memory device, the modulation of *V*_TH_ is required to facilitate the operation of a 1S1R device [17]. Moreover, the large difference between *V*_TH_ and *V*_Hold_ can alleviate the operational complexity of a CPA structure and relax the stringent voltage-matching requirements [18, 19].

The switching mechanism of such selector devices using an active metal, such as Ag or Cu, is based on the formation and dissolution of the metallic conduction channel. Therefore, the matrix of the electrolyte material significantly affects the migration of the active metal and switching speed of the selector. The switching speed of a selector based on an oxide-based electrolyte is generally slower than the order of microseconds [20–22], which is relatively slow when compared with that of previously reported OTS [23] or MIT selector devices [24]. Meanwhile, defects in chalcogenide films, such as nonbonded Te (NBT), can lower the activation energy for the migration of active metal ions; therefore, chalcogenide materials are preferable for the fast migration of active metal ions [18]. However, because of their randomly formed metallic conduction channel, these materials have disadvantages in terms of their switching endurance characteristic, which is a crucial factor for selectors [14, 18, 25]. The endurance of an ECM device can be improved from 10^3^ to 10^6^ cycles using an intermediate buffer layer [26]. However, further endurance improvement is required for practical applications of such devices in CPA structures [5].

In this study, a highly defective amorphous Ga_2_Te_3_ was used as a switching layer by inserting an Ag layer to investigate the TS characteristics in terms of a low leakage current (off-state current), high selectivity, modulation of *V*_TH_ and *V*_Hold_, and high endurance. Amorphous Ga_2_Te_3_ is advantageous as an electrolyte material because there are several NBTs that lower the activation energy of Ag migration and Ga vacancy, which acts as a migration site for Ag in amorphous Ga_2_Te_3_ films [27–29].

## Methods

**Fig. 1:**
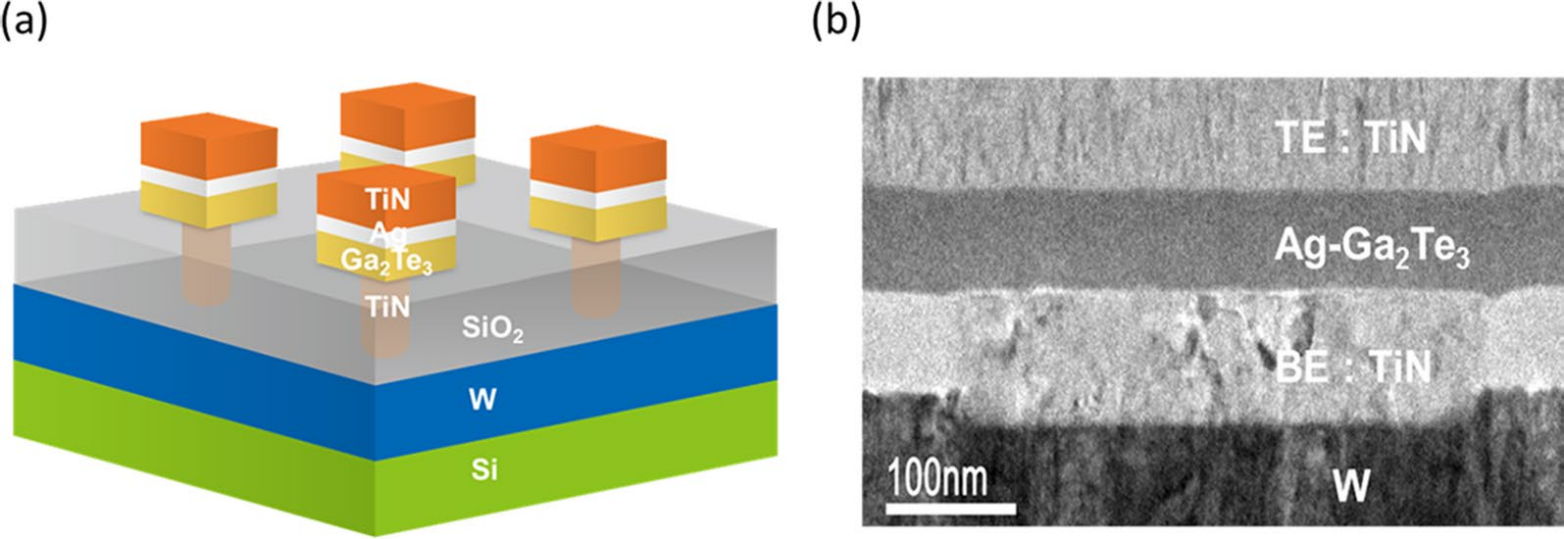
**a** Schematic of the Ag/Ga_2_Te_3_ selector devices. **b** Cross-sectional TEM image of the TiN/Ag-Ga_2_Te_3_/TiN selector device

Selector devices of TiN/Ag/Ga_2_Te_3_/TiN stacks were fabricated with a via-hole structure to investigate their TS characteristics, as depicted in Figure 1a. First, TiN plugs with a size of 0.42 μm × 0.42 μm were formed as the bottom electrodes (BEs). Ga_2_Te_3_ thin films with thicknesses of 40 nm were deposited through RF magnetron co-sputtering using Ga_2_Te and Te targets. Subsequently, an Ag film with a thickness of 10 nm was deposited on Ga_2_Te_3_ films through DC magnetron sputtering. Finally, a TiN top electrode (TE) was formed using DC magnetron sputtering and a lift-off method.

The electrical properties were investigated using a Keysight B1500A analyzer at 298 K. DC switching tests were conducted with a compliance current (*I*_comp_) to avoid the hard breakdown of TS devices. In addition, AC *I*−*V* measurements were conducted with an external load resistance of 1 MΩ to prevent the breakdown of devices. The microstructure of the device was investigated using transmission electron microscopy (TEM; JEOL FEM-F200), as shown in Fig. 1b. The cross-sectional TEM samples of devices were prepared using a focused ion beam system. The atomic distribution of Ag in the Ga_2_Te_3_ film was investigated using TEM-energy dispersive spectroscopy (EDS) measurements.

## Results and Discussion

**Fig. 2:**
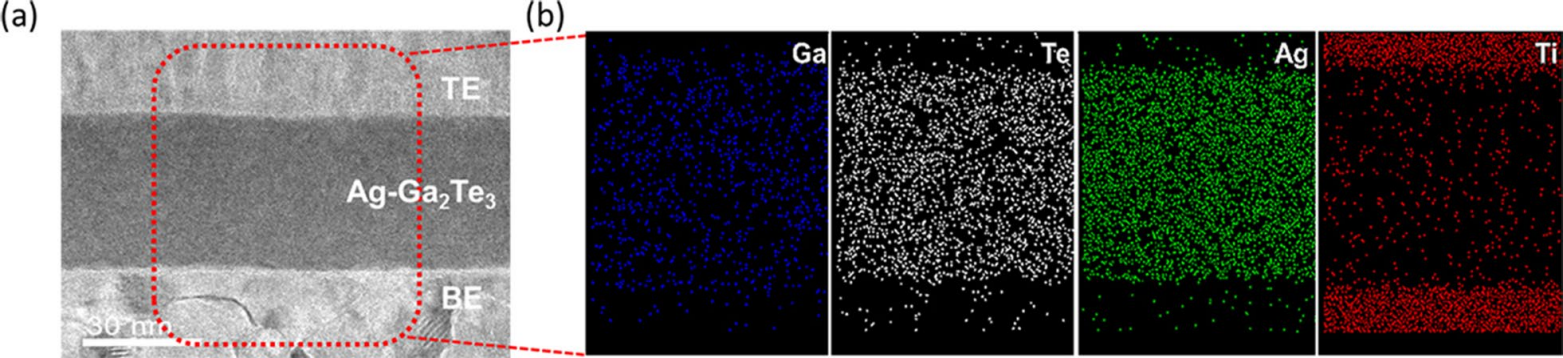
**a** Cross-sectional TEM image of the TiN/Ag-Ga_2_Te_3_/TiN device structure. **b** TEM–EDS mapping images of Ga, Te, Ag, and Ti for the red rectangular region marked in **a**

Figure 2a shows a cross-sectional TEM image of the pristine TiN/Ag-Ga_2_Te_3_/TiN stack of a selector device. The Ag interlayer with a thickness of 10 nm was not observed on top of the Ga_2_Te_3_ thin film. Figure 2b presents the EDS mapping of the Ga, Te, Ag, and Ti elements for the red rectangular region marked in Fig. 2a. The EDS mapping images show that Ag is uniformly distributed in the Ga_2_Te_3_ film even though a co-sputtering process of Ag was not applied. The homogeneous Ag-Ga_2_Te_3_ film may have been formed probably because of the diffusion of Ag during the stack formation. Such fast homogenization of Ag was also reported for the GeTe films [30–32]. Ag may diffuse into the Ga_2_Te_3_ thin film owing to defects such as NBT and Ga vacancies in the Ga_2_Te_3_ thin films [18, 27–29].

**Fig. 3:**
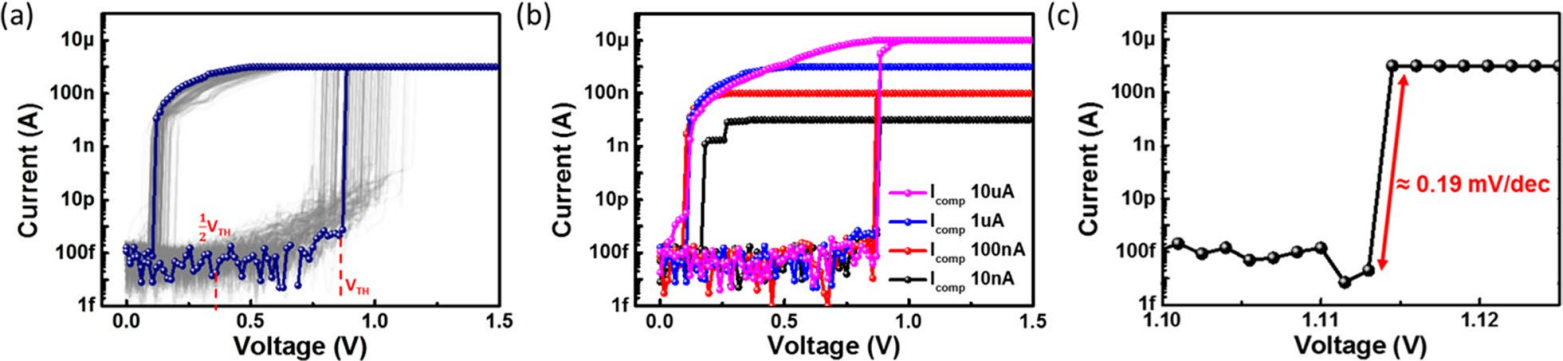
**a***I*–*V* characteristics of the Ag-Ga_2_Te_3_ selector device for DC voltage sweep results during 100 consecutive cycles. The Ag-Ga_2_Te_3_ selector device shows significantly low leakage current (< 100 fA) with an on/off ratio of 10^8^. **b** TS characteristics of the Ag-Ga_2_Te_3_-based selector device at various *I*_comp_ values from 10 nA to 10 μA. **c** Close-up view of the *I*–*V* curve at TS that shows a turn-on slope of 0.19 mV/dec

Figure 3a shows the current−voltage (I−V) characteristics of the Ag-Ga_2_Te_3_ devices with a bottom electrode area of 0.42 µm × 0.42 µm for 100 consecutive cycles of DC sweeps. The device showed TS characteristics without a forming process. When the voltage on TE swept from 0 to 1.5 V, the conduction current increased abruptly at the *V*_TH_ ≈ 0.87 V to *I*_comp_ that was set to 1 µA, which indicated that the device switched from a high-resistance state (HRS) to a low-resistance state (LRS). The device relaxed back to the HRS at *V*_Hold_ ≈ 0.12 V when the voltage was reduced from 1.5 to 0 V, demonstrating a considerable difference between *V*_TH_ and *V*_Hold_. The off-state current at *V*_TH_ was measured to be less than 100 fA, which corresponds to one of the lowest values when compared to previously reported chalcogenide-based selectors using active metals such as Ag or Cu [14, 18, 25, 30, 33]. The selectivity, which is defined as the ratio of the on-state current to the off-state current, was approximately 10^8^. As shown in Fig. 3b, the I−V curves showed stable TS characteristics for various *I*_comp_ values ranging from 10 nA to 10 µA, indicating its flexibility in the operation current. The forming-free TS with a large difference between *V*_TH_ and *V*_Hold_ of the Ag-Ga_2_Te_3_ selector devices are distinctly favorable over the TS characteristics of the Ga_2_Te_3_-only OTS selector devices [34]. Because the forming process is considered as a potential obstacle for real device applications, the forming-free characteristics of the Ag-Ga_2_Te_3_ device are more favorable than selector device, which requires a forming process [35]. Further, the TS characteristic with a large hysteresis of the Ag-Ga_2_Te_3_ selector device may lower the operational complexity of the CPA structure and ease the stringent voltage-matching requirements [18, 19]. Additionally, the Ag-Ga_2_Te_3_ selector shows a steep turn-on slope of 0.19 mV/dec with a scan rate of 1.5 mV per measurement step, as shown in Fig. 3c. The Ag-Ga_2_Te_3_ selector device demonstrated excellent characteristics including its high selectivity (10^8^), low off-state current (<100 fA), steep turn-on slope (0.19 mV/dec), and forming-free characteristics.

**Fig. 4:**
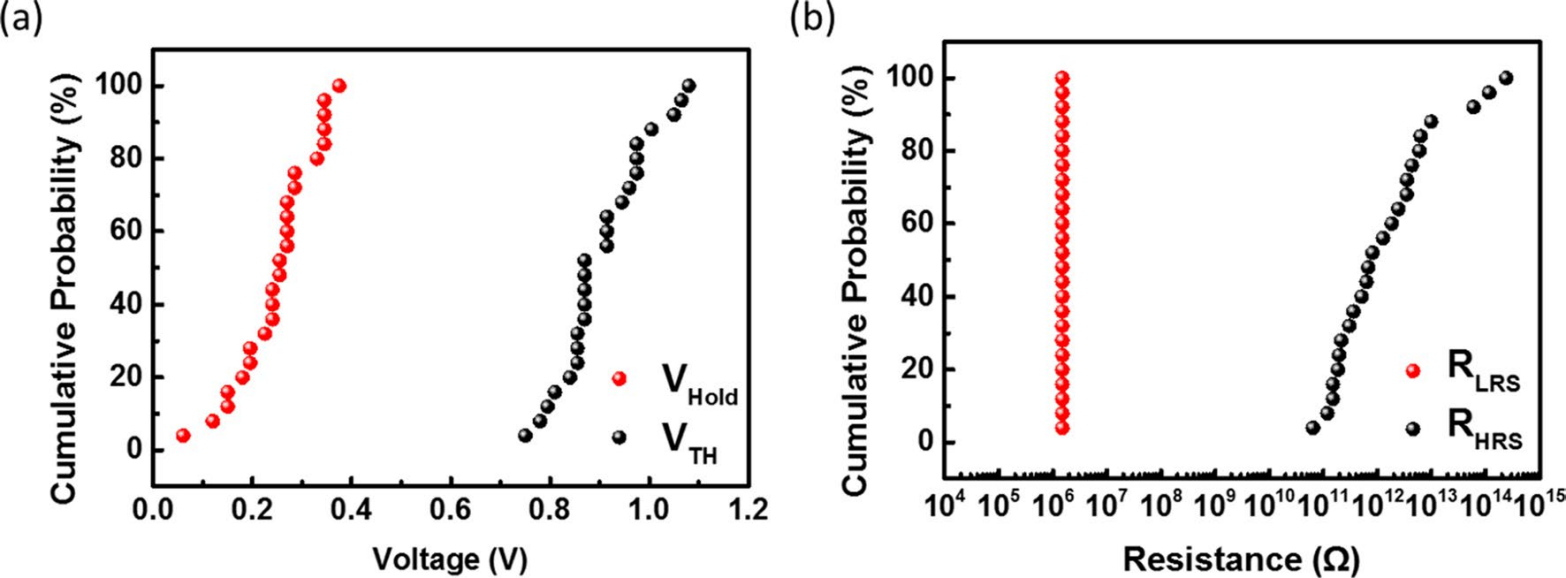
**a** Device-to-device variations of *V*_TH_ and *V*_Hold_ for 25 devices. **b** Device-to-device variations of *R*_HRS_ and *R*_LRS_ for 25 devices

As variation in device performance is a crucial factor for the application of a selector to a CPA structure, the distributions of *V*_TH_, *V*_Hold_, resistance of the high-resistance state (*R*_HRS_), and resistance of the low-resistance state (*R*_LRS_) were investigated for 25 random devices. Figure 4a shows that the distribution of the threshold voltage ranged from 0.75 to 1.08 V, while the hold voltage distribution ranged from 0.06 to 0.375 V. In addition, the resistance distribution at the HRS ranged from 10^11^ to 10^14^ Ω, while the resistance at the LRS was approximately 10^6^ Ω, as shown in Fig. 4b. Owing to the metal conduction channel formation, selector devices using active metals such as Ag or Cu exhibit relatively wide variation characteristics [36, 37]. Accordingly, studies on improving the reliability of these characteristics via doping or buffer layer insertion have been reported [37, 38].

**Fig. 5:**
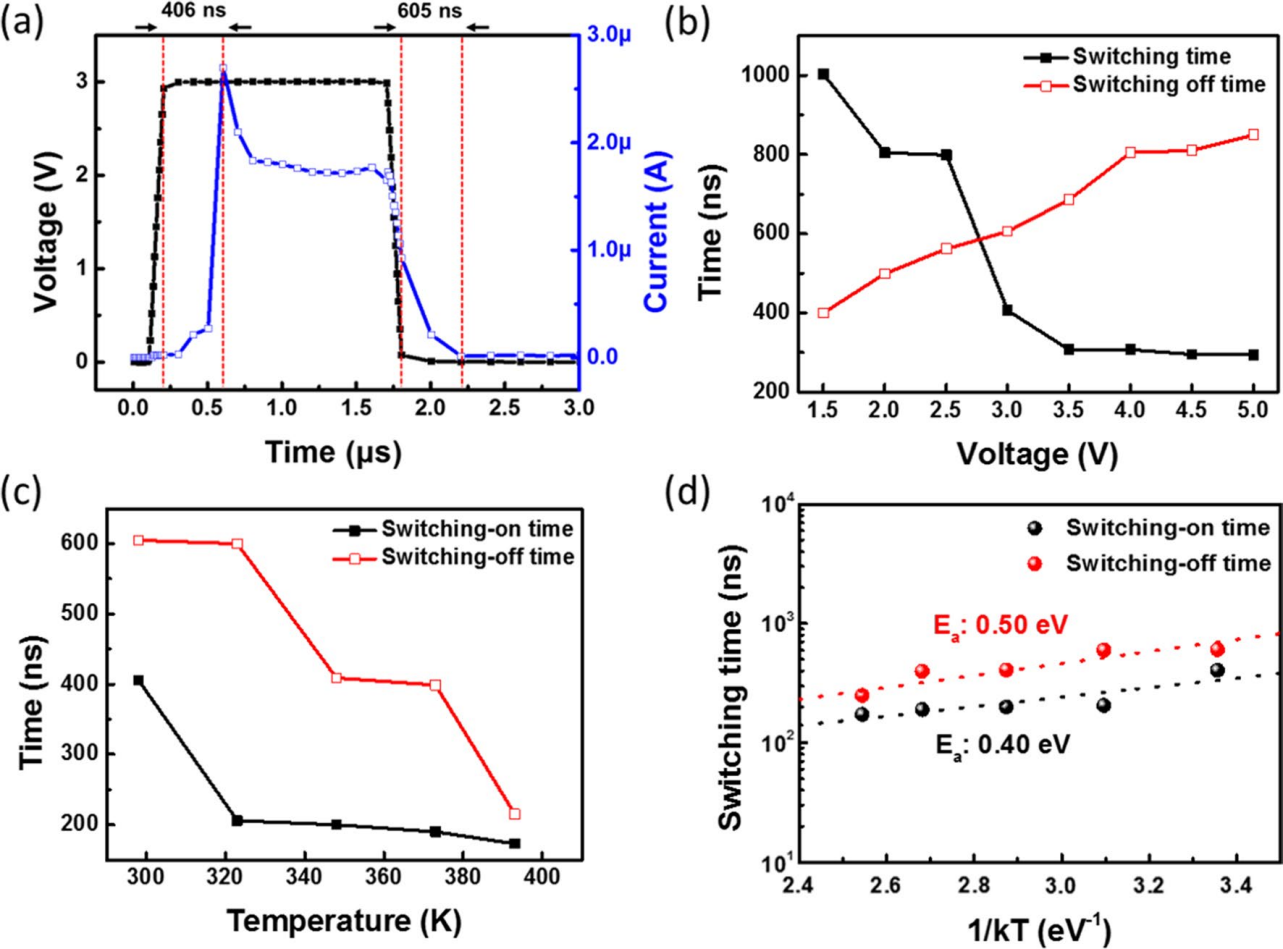
**a** AC *I*–*V* measurement of the Ag-Ga_2_Te_3_ selector device (measurement conditions: rising time = 100 ns, duration = 1.5 μs, falling time = 100 ns, and input voltage = 3 V). **b** Switching speed dependence on applied pulse voltage. **c** Switching speed dependence on measurement temperature. **d** Arrhenius plot of switching speed against measurement temperature

To investigate the transient response of the Ag-Ga_2_Te_3_ selector, the current was measured using a waveform generator fast measurement unit (WGFMU) during a voltage pulse with a height of 3 V, rising−falling time of 100 ns, and duration of 1.5 μs with an external load resistance of 1 MΩ, as shown in Figure 5a. The conduction current of the Ag-Ga_2_Te_3_ selector device reached its peak value after 406 ns from the point at which the voltage reached its maximum of 3 V. Furthermore, the device was switched to the off-state within 605 ns after the applied voltage was removed. Hence, the switching-on time and switching-off time of the Ag-Ga_2_Te_3_ selector were estimated to be approximately 400 ns and 600 ns, respectively. The slow switching of the Ag-Ga_2_Te_3_ selector can be attributed to the migration and redox reactions of Ag for the formation of the conduction channel. In addition, the influence of the applied voltage and measurement temperature on the switching time was investigated with an input voltage of 1.5−5 V and at a measurement temperature of 298−375 K. The switching-on time was decreased from 1 μs to 294 ns, whereas the switching-off time was increased from 400 ns to 849 ns as the pulse voltage was increased from 1.5 to 3.5 V, as shown in Fig. 5b. The dependence of the switching speed on the applied voltage is comparable with the previously reported results of Ag layer on HfO_2_ and TiO_2_ [39]. Moreover, Fig. 5c shows that the switching-on and switching-off times decreased with increasing measurement temperature. According to the Arrhenius plot of switching speed against measurement temperature shown in Fig. 5d, the exponential dependence of switching speed on measurement temperature can be attributed to thermally facilitated processes, such as the diffusion of Ag atoms in the electrolyte film matrix [40]. The activation energies for switching-on and switching-off were estimated to be 0.50 eV and 0.40 eV, respectively, which are comparable with those presented in a previous report on a Ag filament-based device [41]. It was reported that the Ag conductive channels were formed under electrical bias in HfO_2_, SiO_2_, and TiO_2_ [15, 42, 43]. However, in this study, Ag was observed to be uniformly distributed in pristine Ga_2_Te_3_ films. Although the mechanism for TS in Ga_2_Te_3_ films with uniform distribution of Ag is not clearly understood, Ag may be related to the formation of conductive channels in Ga_2_Te_3_ films under electrical bias. Therefore, the dependence of switching speed on the input voltage and measurement temperature of the Ag-Ga_2_Te_3_ selector device can be attributed to the formation of the conductive channels.

**Fig. 6:**
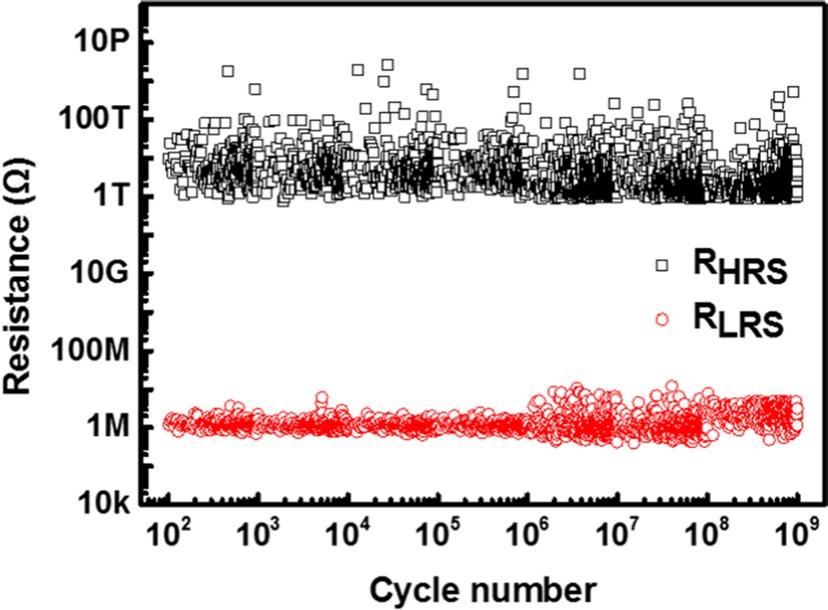
AC endurance characteristic of the Ag-Ga_2_Te_3_ selector device up to 10^9^ cycles (0.5 V and 3 V reading voltages for *R*_HRS_ and *R*_LRS_, respectively)

The AC endurance characteristic was investigated under the same voltage pulse condition as that of the switching speed test. The reading voltages for the HRS and LRS were 0.5 and 3 V, respectively. The measured resistances of the HRS and LRS were plotted for 450 points per decade, as shown in Fig. 6. The Ag-Ga_2_Te_3_ selector device exhibited stable endurance characteristics up to 10^9^ cycles maintaining a selectivity of 10^8^, thus demonstrating excellent switching endurance characteristics when compared with those of other selectors that utilized chalcogenide and active metals [18, 25, 30].

## Conclusions

In this study, we demonstrated the stable TS characteristics of a selector device fabricated using Ag with high ion mobility and highly defective amorphous Ga_2_Te_3_ as a switching layer. TEM analyses of the TiN/Ag-Ga_2_Te_3_/TiN structure showed that the embedded Ag interlayer was completely diffused into the Ga_2_Te_3_ film to produce uniform Ag distribution in the Ga_2_Te_3_ layer. This may be because of the highly defective structure of amorphous Ga_2_Te_3_ during subsequent TE TiN deposition. The Ag-Ga_2_Te_3_ selector device exhibited forming-free TS, a large hysteresis (1 V), high selectivity (10^8^), low off-state current (<100 fA), steep turn-on slope (0.19 mV/dec), and excellent endurance characteristics (10^9^ cycles). In addition, AC I−V measurements showed the switching speed to be in the order of hundreds of nanoseconds. The dependence of switching speed on pulse voltage may be the combined effect of Ag migration and redox reaction. Moreover, the Arrhenius behavior of switching speed based on the measurement temperature suggested that the TS is related to a thermally facilitated process. In conclusion, the Ag-Ga_2_Te_3_ device with the excellent TS and endurance characteristics is a promising candidate for selector in the CPA memory applications.

## Data Availability

All data are fully available without restriction.

## Abbreviations

3D: 3-Dimensional
CPA: Cross-point array
TS: Threshold switching
OTS: Ovonic threshold switch
MIT: Metal–insulator transition
FAST: Field-assisted super-linear threshold switch
ECM: Electrochemical metallization
MIEC: Mixed-ionic-electronic conduction
*V*_TH_: Threshold voltage
*V*_Hold_: Hold voltage
1S1R: One selector-one resistor
*V*_set_: Set voltage
NBT: Non-bonded Te
TE: Top electrode
BE: Bottom electrode
*I*_comp_: Compliance current
HRS: High-resistance state
LRS: Low-resistance state
*R*_HRS_: Resistance of the high-resistance state
*R*_LRS_: Resistance of the low-resistance state
